# Modified HEK cells simulate DCT cells in their sensitivity and response to changes in extracellular K

**DOI:** 10.14814/phy2.14280

**Published:** 2019-11-24

**Authors:** Meena Murthy, Kevin M. O’Shaughnessy

**Affiliations:** ^1^ Division of Experimental Medicine and Immunotherapeutics Department of Medicine University of Cambridge Cambridge UK

**Keywords:** chloride sensing domain, NCC, potassium, SPAK kinase, WNK kinases

## Abstract

A potassium (K^+^) rich diet is known to have an antihypertensive effect that has been embodied by the NHLBI in the DASH diet. However, the molecular basis for this blood pressure‐lowering effect has been unclear, until a recent study proposed a model in which the DCT cells of the kidney regulate their salt transport in response to variations in intracellular chloride ([Cl^−^]_i_), which are directly regulated by serum K^+^. With the knowledge that WNK proteins are Cl^−^ sensors, and are a part of the WNK/SPAK/NCC signaling cascade which regulates the NCC, the main salt transporter in the distal nephron, we examined the effect of serum K^+^ on the ([Cl^−^]_i_) and, in turn its effect on the WNK4 signaling pathway in a “modified HEK 293T” cell line. Using a fluorescence‐based approach in this cell line, we have shown that the membrane potential of the cell membrane is sensitive to the small changes in external KCl within the physiological range (2–5 mM), thus functioning as a K^+^ electrode. When the extracellular K^+^ was progressively increased (2–5 mM), the membrane depolarization lead to a subsequent increase in [Cl^−^]_i_ measured by fluorescence quenching of an intracellular chloride sensor. Increase in extracellular [K] resulted in a decrease in the phosphorylation of the WNK4 protein and its downstream targets, SPAK and NCC. This confirms that small changes in serum K can affect WNK4/SPAK/NCC signaling and transcellular Na^+^ flux through the DCT and provide a possible mechanism by which a K‐rich DASH diet could reduce blood pressure.

## Abstract 2

We have tested the Ellison model directly to show that the effect of plasma [K^+^] on NCC levels is mediated by changes in [Cl^−^]I, in response to changes in the V_m_ of the cell. To do this we have used an in vitro system, i.e., modified HEK‐293T cells which simulate DCT cells generated by transient transfection of the necessary ion channels/transporters, including KCNJ10, Clc‐kb and Barttin, typically found in DCT cells. A fluorescence based approach was has been used to show that increasing [KCl]_o_ depolarizes the cells and causes inactivation of the WNK/SPAK/NCC cascade.

## Graphical abstract

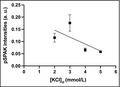

## 1 INTRODUCTION

Hypertension is a major public health issue among industrialized nations, and several clinical and epidemiological studies have shown a clear link between dietary Na^+^ intake and blood pressure. With Na^+^ and K^+^ homeostasis being closely interlinked, a low Na^+^ and high K^+^ diet has been shown to lower blood pressure (Mente et al., 2014). Studies on monogenic syndromes such as Gordon's (Familial Hyperkalaemia & Hypertension, http://omim.org/entry/145260) and Gitelman (OMIM http://omim.org/entry/263800) have shown that mutations in ion channels and transporters and/or their regulators such as WNK proteins in the nephron perturb the Na^+^/K^+^ homeostasis and cause significant changes in blood pressure. The distribution of these proteins along the nephron reflects the key importance of these nephron segments including the loop of Henlé (LoH), the distal convoluted tubule (DCT), and the contiguous segments of the connecting tubule (CNT) and collecting duct (CD) in the homeostatic process. The WNK/SPAK pathway regulates various ion transporters/channels in this segment of the nephron, and thus WNK kinase expression and activity is pivotal. In the DCT, WNKs are activated by sympathetic stimulation, cortisol, (Terker et al., 2014) low extracellular K^+^, (Terker et al., 2015) and low intracellular Cl^−^ (Piala et al., 2014; Terker, Zhang, et al., 2016), which affect its phosphorylation state that in turn leads to an activation of downstream targets. WNK kinases phosphorylate and activate SPAK (STE20/SPS1‐related proline‐alanine‐rich protein kinase) and OSR1 (Oxidative Stress Responsive 1), which in turn bring about the coordinated phosphorylation of NCC and NKCC2 in the DCT and TAL of the nephron, respectively (Richardson & Alessi, 2008; Vitari et al., 2006). In the DCT, the WNKs are degraded by the proteasome pathway when they are targeted by a kelch‐like protein 3 (KLHL3) mediated ubiquitylation by Cullin3 (Cul3) (Ohta et al., 2013; Schumacher et al., 2015).

Studies on the WNK/SPAK/NCC signaling pathway have elucidated the molecular link between WNK kinases and the regulation of blood pressure by their effect on renal electrolyte homeostasis, and how the aldosterone regulated the processes of Na^+^ absorption (from the urine to the blood) and K^+^ secretion (from the blood to the urine) work in concert in the distal nephron (Kahle et al., 2003). Although the benefits of a high‐K diet in lowering blood pressure has been well‐established, only the recent finding that WNK kinases are chloride‐sensitive has provided a clue to the molecular basis for this effect (Ellison & Terker, 2015). WNK kinases switch between active and inactive states based on their phosphorylation status, with phosphorylation stabilizing the active state. Cl^−^ ions stabilize the inactive WNK1, and this prevents any kinase autophosphorylation and activation, thus explaining how the changes in intracellular chloride and tonicity affect the WNK kinase activity (Piala et al., 2014). This activation is rapid, occurring in less than 0.5 min, and is due to the phosphorylation of the serine residue in the T‐loop which is conserved across all the WNKs. Crystallographic data on WNK1 have shown that it has an “LGL” motif, also called a “chloride sensor,” that confers chloride sensitivity by blocking the autophosphorylation of the T‐loop, and thus confirming that WNKs are the key chloride sensing kinases which regulate the behavior of Na^+^ and K^+^ cation cotransporters such as NCC and NKCC2 (Haas & Forbush, 2000). Mutating the leucine residue in the LGL motif (LL to FF) causes an activation of WNK kinase function which is most pronounced in WNK4 (Piala et al., 2014). Recently, Ellison has proposed a model for DCT function in which low external K^+^ ([K^+^]_o_) causes the DCT cell to hyperpolarize, resulting in the efflux of Cl^−^, a reduction in [Cl^−^]_i_ and activation of the WNK/SPAK/NCC pathway. With high [K^+^]_o_, the contrary occurs–the DCT is depolarized, Cl^−^ enters the cell and the rise in [Cl^−^]_i_ deactivates the WNK4/SPAK/NCC pathway (Terker et al., 2015). Thus the level of Na^+^ reabsorption at the DCT is directly regulated by the [K^+^]_o_, which determines the phosphorylation state of NCC by controlling the activity of the WNK/SPAK/NCC pathway.

We have tested the Ellison model directly to show that the effect of plasma [K^+^] on NCC levels is mediated by changes in [Cl^−^]_i,_ in response to changes in the membrane potential (V_m_) of the cell. To do this, we have used an in vitro system, that is, modified HEK‐293T cells generated by transient transfection of the necessary ion channels/transporters, including KCNJ10, Clc‐kb, Barttin, typically found in DCT cells. A fluorescence‐based approach was used to directly show that increasing [K^+^]_o_ depolarizes the cells and causes subsequent inactivation of the WNK/SPAK/NCC cascade.

## 2 MATERIALS AND METHODS

### 2.1 Cell culture and transfection

Human embryonic kidney HEK‐293 T cells were cultured in DMEM high glucose supplemented with 10% (v/v) fetal bovine serum, 2 mM L‐glutamine, 100 units/ml penicillin, and 0.1 mg/ml streptomycin at 37°C in 5% CO_2_–95% air atmosphere. For transfection experiments, cells were grown on 10 mm petri dishes and were incubated for a period of 36 hr with a mixture of 20 μL of 1 mg/ml polyethylenimine (Polysciences) and 5–10 μg of the respective plasmid DNA (KCNJ10, Clc‐kb, and barttin from Origene), as described (Durocher, Perret, & Kamen, 2002). Depending on whether the V_m_ or [Cl^−^]_i_ was going to be measured in the cells, the plasmid constructs pcDNA3.1 (−) VSFP3.1 (Addgene) or pcDNA3.1 (+) EYFP H148Q/I152L (Addgene) were transfected into the cells. Transfected cells were then transferred to poly‐L‐lysine coated 96‐well, black microplates (Greiner bio‐one), and allowed to adhere overnight before use. Simultaneous measurements of both V_m_ and [Cl^−^]_i_ in cells by co‐expression of the voltage and Cl sensor were not feasible because of the large overlap of the two emission spectra of CFP and EYFP.

### 2.2 Measurement of V_m_, and [Cl^−^]_i_ using the Flex station microplate reader

HEK cells transfected with the K^+^ channel *KCNJ10*, and the voltage‐sensitive dye VSFP3.1 (encoded by pcDNA3.1 (−), Addgene) were used to measure the change in V_m_ at an absorption and emission wavelengths of 444 and 535 nm, respectively. HEK cells transfected with the chloride sensor, pcDNA3.1 (+) EYFP H148Q/I152L (Addgene), *KCNJ10, Clc‐kb* and *barttin* were used to measure the change in [Cl^−^]_i_ at an absorption and emission wavelengths of 485 and 555 nm, respectively.

Immediately before the Flex station measurements, 96‐well plates with the adhered HEK cells were washed with a solution containing 130 mM NaCl, 10 mM NaI, 2 mM KCl, 2 mM CaCl_2_, 1 mM MgCl_2_, and 20 mM HEPES pH 7.3. The cells were then incubated in 100 µl of the same solution at 37°C for 45 min and the fluorescence was measured in a FlexStation 3 microplate reader (Molecular Devices) at 2 s intervals for 200 s. NaCI was included only in the solutions which were used for [Cl^−^]_i_ measurement. Solutions with different external KCl concentrations were introduced to the cells in each well after 50s of baseline measurement (the concentration of NaCl was adjusted in each case to maintain the osmolality of the solution). Appropriate negative controls were employed, and selective blockers including DIDS (Sigma) (L'Hoste et al., 2013), Valsartan (Sigma) (Imbrici et al., 2017), Tertiapin Q (Abcam) (Doupnik, 2017), and Ba (Sigma) were used as indicated. The change in fluorescence (Δ*F*) was defined as *F*_max_ (peak fluorescence) – *F*_min_ (baseline fluorescence). EC_50_ values were obtained by fitting data to four‐variable concentration–response curves using Prism 5 (GraphPad Software Inc., San Diego, CA).

### 2.3 Biochemcial studies

HEK‐293 T cells were grown in normal cell culture conditions, and then transfected with mouse *NCC* (pECFP‐C1‐NCC) alone, or with *WNK4 WT*, *WNK4 L322F L324F* mutant or the *WNK4 LLFF* kinase‐dead mutant form respectively (in pMO‐Myc vector, a generous gift from Prof D. Ellison, University of Oregon), and grown in medium with different external KCl concentrations (2–5 mM).

### 2.4 Biotinylation

Transfected HEK 293T cells in 10 cm plates were biotinylated following a previously published method (Murthy, Kurz, & O'Shaughnessy, 2016) Briefly, HEK 293T cells in 10 cm plates were washed once in NES buffer and placed on ice. Cells were incubated in 0.2 mg/ml NHS‐ biotin (www.thermofisher.com) for 45 min on ice before quenching with Tris buffer (25 mM Tris‐HCl; 150 mM NaCl; 10 mM EDTA, pH 7.4). Cells were removed from the plate and centrifuged at 200*g* for 5 min at 4°C. Supernatant was discarded and cells were incubated in solubilization buffer (25 mM Tris‐HCl; 150 mM NaCl; 10 mM EDTA; 1% Triton X‐100, pH 7.4) with protease inhibitors (www.roche.co.uk) at 4°C with agitation for 1 hr. The resulting lysate was cleared by centrifugation at 14 K rpm 4°C for 1 hr. Thirty microliters of streptavidin beads (www.thermofisher.com) per sample were washed once in wash buffer (25 mM Tris‐HCl; 150 mM NaCl; 10 mM EDTA; 1% Triton X‐100, pH 7.4), centrifuged at 1,400 rpm for 5 min at 4°C and supernatant removed. The supernatant was added to streptavidin beads and incubated with agitation for 2 hr at 4°C. Beads were then pelleted at 14K rpm for 5 min at 4°C and washed three times in wash buffer before resuspending the pellet in 4× sample buffer (www.lifetechnologies.com) for Western blotting.

### 2.5 Western blotting

For blots with nonbiotinylated cell lysates, cells were harvested 36 hr post‐plasmid transfection from the 10 cm plates, and lysed in 300 µl lysis buffer (Terker, Zhang, et al., 2016) (supplemented with protease inhibitors, phosphatase inhibitors, and 1 mM DTT). After protein quantification by Bradford's method, 20 µg equivalent total protein lysate was loaded on to 3%–8% Tris‐acetate Nupage gel and transferred to nitrocellulose membranes. Membranes were Ponceau stained to check for transfer efficiency and loading, then blocked with 5% milk for 1 hr and incubated with primary antibody in 5% (w/v) skimmed milk in TBS‐T with the indicated primary antibodies: 1 in 500 diluted rabbit phospho‐WNK4 Ser575 antibody (a generous gift from Prof Isao Naguro, Japan) or 2 µg/ml sheep phospho‐SPAK Thr373 antibody and peptide (to prevent binding to nonphosphorylated SPAK (10 µg/ml) (Division of Signal Transduction Therapy Unit, University of Dundee) or pNCC Thr58 (a generous gift from Prof J Loffing, University of Zurich) (1 in 1,000 dilution) overnight at 4°C. β‐actin (mouse antibody from Thermo Fisher, 1 in 2,000 dilution) was stained as a loading control. The blots were then washed three times with TBS‐T and incubated in secondary antibodies, donkey anti rabbit 800CW (926–32213 at 1:5,000 dilution; www.licor.com), and goat anti mouse Alexa680 (A‐21058 at 1:5,000 dilution; www.lifetechnologies.com) in TBS‐Tween 20 for 1 hr at room temperature in the dark and then washed six times in TBS‐Tween 20. Membranes were imaged using the LiCor Odyssey system (www.licor.com), and the blots were quantified using the Image studio Lite software www.licor.com).

### 2.6 Statistical analysis

All data are shown as mean ± *SEM* unless indicated otherwise. Significance was determined using unpaired *t* test or one‐way ANOVA followed by post hoc testing as appropriate. *p* < .05 was considered significant. The stats package in Prism4 (http://www.graphpad.com/scientific-software/prism/) was used.

## 3 RESULTS

### 3.1 Measurement of change in membrane potential (V_m_)

**Figure 1:**
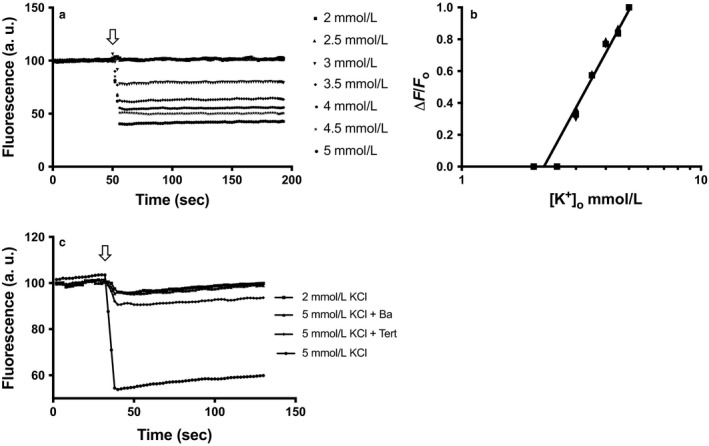
HEK cells transfected with *VSFP3.1* and *KCNJ10* were exposed to different [KCl]_o_. Fluorescence at 2 mM KCl recorded from 1 to 200 s was adjusted to a baseline of 100%. For those wells where [KCl]_o_ was changed, a baseline was obtained for the first 50 s in 2 mM KCl, after which the [KCl]_o_ was increased from 2 through 5 mM KCl in 0.5 mM steps, respectively (a). CFP quenching at different [KCl]_o_. (b) Normalized response to KCl (Δ*F*/*F*o) plotted semilogarithmically against final [K]o. Data points are means of triplicates. (c) Effect of K channel blockers on CFP quenching

Cells transfected with the voltage‐sensitive VSFP3.1 exhibited a typical CFP (cyan fluorescent protein, emission max at 535 nm) emission spectrum that was sensitive to membrane depolarization when the [KCl]_o_ was changed progressively from 2 through 5 mM KCl (Figure 1a). This effect of increase in external K^+^ resulted from increasing quenching of the CFP fluorescence (Figure 1a) over the range of [KCl]_o_ used, the normalized change in fluorescence (ΔF/Fo) exhibited a log‐linear relationship with increasing external KCl (Figure 1b), showing that the membrane functions as a K^+^ electrode. The fluorescence quenching in 5 mM [KCl]_o_ was affected by the presence of the K channel blockers Ba^2+^ or Tertiapin Q (Figure 1c); Ba^2+^ abolished the quenching almost completely, while Tertiapin Q (50nM)`reduced it to about 10%. The quenching response to increasing external KCl was not observed in cells, which were not transfected with *KCNJ10* (data not shown).

### 3.2 I^‐^ affects basal YFP quench

YFP, a genetically modified variant of green fluorescent protein (GFP), is halide sensitive, and the H148Q/I152L mutant form of YFP has increased sensitivity to halides with the relative affinity of anions to YFP‐H148Q/I152L being I^−^>NO_3_^−^>Br^−^> Cl^−^ (Galietta, Haggie, & Verkman, 2001). About 10 mM NaI was used in all our experimental solutions, based on previous work with this fluorophore (Johansson, Norris, & Peilot‐Sjogren, 2013) showing that 10 mM NaI was an optimal concentration for assay performance while ensuring that there is no artifact of agonist‐independent I^‐^ permeability of the cells. EYFP quenching was not observed when cells were assayed in the absence of I^‐^ (data not shown).

### 3.3 Changing external [KCl] affects EYFP fluorescence in the cells

**Figure 2:**
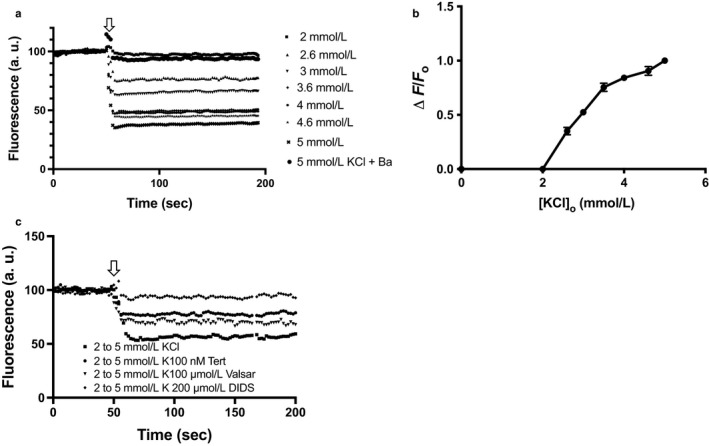
HEK cells transfected with *EYFP H148Q/I152L*, *KCNJ10, Clc‐kb,* and *barttin* and exposed to different [KCl]_o_. Fluorescence at 2 mM KCl recorded from 1 to 200 s was adjusted to a baseline of 100%. For those wells where [KCl]_o_ was changed, a baseline was obtained for the first 50 s in 2 mM KCl, after which the [KCl]_o_ was increased from 2 mM through 5 mM KCl, respectively. (a) EYFP quenching at different [KCl]_o_. (b) Normalized response to KCl (Δ*F*/*F*o) plotted against final [Ko]. Data points are means of four different experiments. (c) Effect of different channel blockers, such as Ba^2+^ (1 mM), Tertiapin Q (100 nM), Valsartan (100 µM), DIDS (200 µM) on EYFP quenching

HEK cells expressing YFP‐H148Q/I152L showed a step‐wise fluorescence quenching in response to increasing [KCl]_o._ A maximal EYFP fluorescence quenching of over 50% was seen when the concentration of KCl was stepped up from 2 to 5 mM, a physiologically relevant range of [K^+^]_o_ (Figure 2a). When the normalized change in fluorescence (Δ*F*/*F*o) was plotted against [K^+^]_o_, the data fitted a four‐variable dose–response curve, with an IC50 of 2.9 mM KCl (Figure 2b). The quenching response to increasing external KCl was not observed in cells which were not transfected with *Clc‐kb* and *barttin* (data not shown).

In the presence of the Cl channel blockers, the fluorescence quenching was substantially reduced. DIDS (4,4^′^‐Diisothiocyanatostilbene‐2,2=‐ disulfonic acid), an inhibitor of Cl^−^ flux (Wulff, 2008), abolished the EYFP quenching almost completely, suggesting that the K effect on Cl^−^ influx and intracellular EYFP depends on the expressed Clc‐kb channel, whereas Valsartan (Imbrici et al., 2017) was less effective in comparison to DIDS. Tertiapin Q also reduced the EYFP quenching by about 50%, suggesting that K^+^ influx into the cells through KCNJ10 was necessary for the subsequent Cl^−^ influx, and EYFP quenching (Figure 2c).

### 3.4 Extracellular KCl affects phosphorylated WNK4 (pWNK4) levels in NCC and WNK4 transfected cells

**Figure 3:**
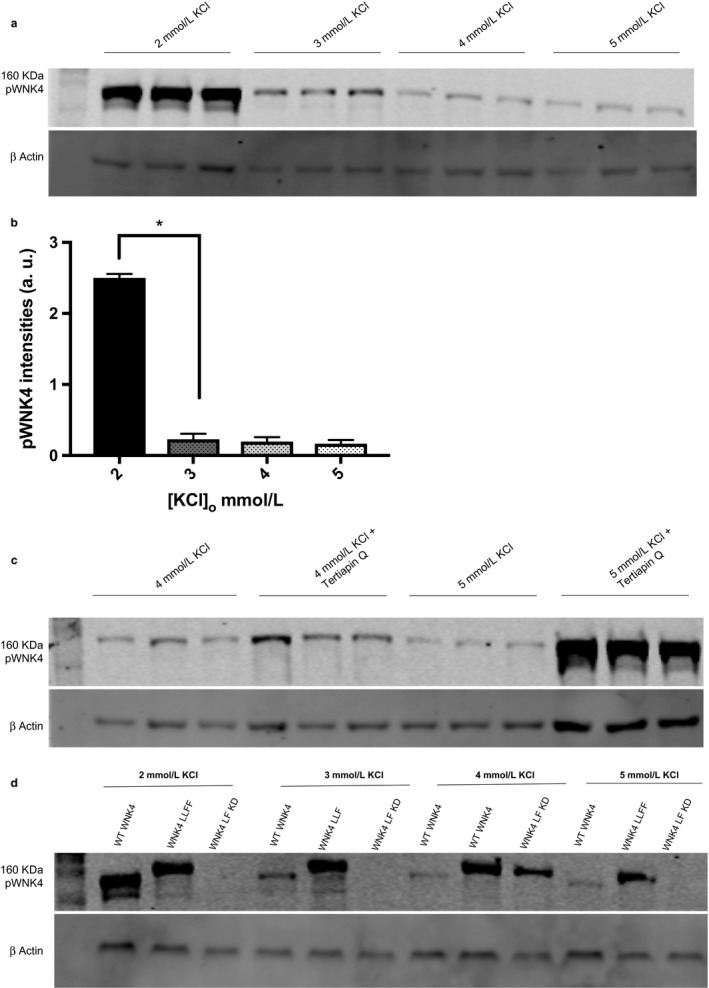
Effect of [K]_o_ on WNK4 phosphorylation in HEK 293 T cells transfected with *NCC* alone or different forms of *WNK4*: *WNK4 WT, WNK4 LLFF*, and *WNK4 LLFF KD*, respectively. (a) Total protein lysates were blotted from HEK 293 T cells transfected with *NCC* and the WT *WNK4* at four different external KCl. Rabbit phospho WNK4 Ser 575 antibody detected a 150 KDa WNK4 band. ß‐actin was the loading control. This is representative of Western blots replicated three times with cell lysates from three different passage numbers. (b) Quantification of Western blots showing phospho WNK4 levels normalized to its ß‐actin loading control. Error bars represent mean ± *SEM* of Western blots (*n* = 3). *p < .0001 by two‐tailed *t* test. (c) The effect of K channel blocker, Tertiapin Q on phospho WNK4 levels at 4 mM, and 5 mM KCl, respectively. (d) Phospho WNK4 levels in cells transfected with the three different WNK4 forms, and at four, different [K]o

Having observed the sensitivity of the “model HEK cell” membrane to coupled changes in [KCl]_o_ and transmembrane Cl^−^ movement, the consequence for the cellular WNK4/SPAK/NCC signaling pathway was investigated. Altering [KCl]_o_ had distinct effects on the phosphorylation status of the different forms of WNK4 in the transfected cells. WNK4 WT was affected inversely with pWNK4 levels falling (Figure 3a) when [KCl]_o_ was increased from 2 to 3 mM and above. A steep fall in pWNK4 levels was observed upon changing the [KCl]_o_ from 2 to 3 mM with a further small reduction in its levels between 3 and 5 mM KCl (Figure 3b). The K channel blocke,r Tertiapin Q, abolished this effect on WNK4 WT levels (Figure 3c), demonstrating the role of the KCNJ10 channel in mediating the effect of changing [K^+^]_o_ on WNK4 levels. Increasing [K^+^]_o_ did not affect the intensities of the Cl insensitive mutant WNK4 LLFF (Figure 3d), confirming the role of the Cl binding domain of WNK4. Finally replacing WT WNK4 with the WNK4 LF kinase‐dead markedly reduced or abolished p WNK4 levels showing the importance of the kinase domain for the changes seen in pWNK4.

### 3.5 Increase in external KCl affects the downstream phosphorylation targets of WNK4

**Figure 4:**
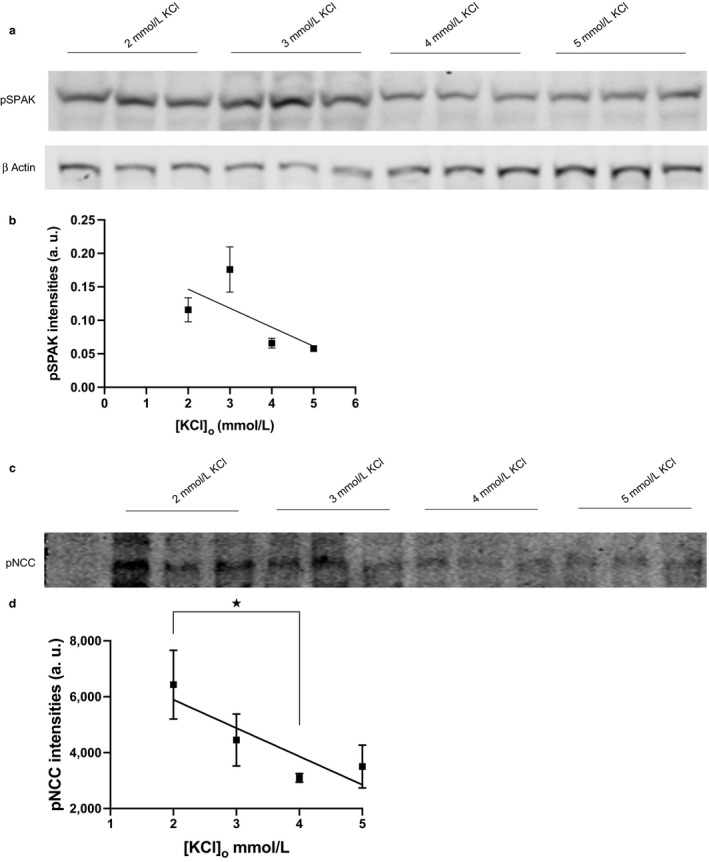
Effect of [K]o on NCC phosphorylation in HEK 293 T cells transfected with *NCC* alone or with *WNK4 WT.* The transfected cells were treated with external KCl concentrations of 2, 3, 4, and 5 mM respectively. Cells were biotinylated. (a) Nonbiotinylated fraction of the cell lysates were blotted. Sheep pSPAK t373 antibody detected an approximately 60 KD band. (b) Quantification of Western blots showing phospho pSPAK levels normalized to its ß‐actin loading control. Error bars represent mean ± *SEM* of Western blots (*n* = 3). *p* < .05 by one‐way ANOVA. (c) Biotinylated protein lysates were blotted and rabbit pNCC Thr 58 antibody detected a 150 KDa phospho NCC band. (d) Quantification of Western blots showing biotinylated phospho NCC levels. Error bars represent mean ± *SEM* of Western blots (*n* = 3). *p < .05 by two‐tailed *t* test

The inverse relationship between [KCl]_o_ and pWNK4 levels was reflected in its downstream phosphorylation targets: SPAK and NCC. Both pSPAK (Figure 4a) and pNCC (Figure 4c) levels decreased with increasing [KCl]_o_ in the model HEK cells expressing WNK4 WT. This fall in pSPAK levels with an increase in [KCl]_o_ from 2 to 5 mM (Figure 4b) possibly explains the decline in the phosphorylation of NCC at a similar range of concentrations of [KCl]_o._ The levels of pNCC is a linear function of [KCl]_o_ within the range of 2 to 5 mM_,_ and the slope of the trendline is comparable (Figure 4d) to the one observed when the pNCC levels were plotted against a similar range of plasma [K^+^] (in the mice untreated with aldosterone) in the in vivo study (Terker, Zhang, et al., 2016). With pNCC being a marker for NCC activity (Pacheco‐Alvarez et al., 2006), the effect of [KCl]_o_ on pNCC suggests that the NaCl entry via NCC would be reduced at physiologically viable higher [K^+^]_o_ due to its restricted activity.

## 4 DISCUSSION

Although K^+^ is the most abundant exchangeable cation in the human body, with an intracellular concentration of 140–150 mM, the serum [K] is normally maintained within a smaller physiological range, 3.5–5 mM. Earlier animal studies have shown that a large increase in dietary K^+^ affects the abundance and activity of the cotransporter NCC (Terker et al., 2015; Vitzthum et al., 2014); however, a recent paper (Terker, Zhang, et al., 2016) has demonstrated that K^+^ is a pivotal modulator of NCC at a physiologically relevant concentration, and proposed a possible mechanism (“Ellison hypothesis”) to explain this. Our in vitro study is a working model of this hypothesis, where the physiological and molecular effects of changing [K^+^]_o_ on intracellular Cl and the WNK/SPAK/NCC pathway (involved in regulating salt transport across the DCT) has been shown in a “DCT‐like” HEK cell system.

Using a CFP fluorescence‐based approach in the model HEK cell system, we have shown that the cell membrane functions as a K^+^ electrode which responds to small, step‐wise changes of 1 mM KCl when the [K^+^]_o_ is increased from 2 mM through 5 mM in cells expressing *KCNJ10*. Significant reduction in EYFP fluorescence was detected as [KCl]_o_ was increased, indicating an inverse relation of EYFP fluorescence quenching to increased [K^+^]_o_. Blockers such as Tertiapin Q and BaCl_2_ abolished these effects of changing [K^+^]_o_, demonstrating the role of the K channels on the DCT‐like cell membrane (including KCNJ10), whereas the ClC channel blockers affected EYFP quenching which is a consequence of Cl^−^ influx across the membrane, and binding of these anions to the EYFP protein. Thus our model cells mimics the hypothesized behavior of DCT cells and confirms they can respond to small changes in [K^+^]_o_ within the physiological range followed by transmembrane anion transport through ClCs.

The model HEK cells expressing WNK4 or its mutant forms respectively responded differently to altering [K^+^]_o_ in the physiologically relevant range of 2 to 5 mM. The [K]_o_ affected the activity of WNK4/SPAK/NCC signaling and its downstream targets in this model cell line as determined by measuring the levels of pWNK4, pSPAK, and pNCC level, respectively. The inverse effect of increasing [K^+^]_o_ on pWNK4 levels in cells expressing wild‐type WNK4 was abolished by K channel blockers (tertiapin and Ba^2+^), thus confirming that DCT K channels (such as KCNJ10) are the route for K entry into these cells, which facilitates the subsequent effects on the WNK4/SPAK/NCC pathway as observed in in vivo studies (Wang et al., 2018). The absence of a similar K^+^ sensitivity to the phosphorylation states of the same proteins in cells expressing the Cl^−^ insensitive mutants, WNK4 LLFF and WNK4 LF KD respectively, emphasises the importance of the WNK4 Cl^−^ binding and kinase domains for transducing the membrane potential changes caused by altered [K^+^]_o_.

WNK4 has been shown to be the most responsive WNK family member to changes in [Cl^−^] (Terker, Zhang, et al., 2016), so it appears to be the key sensor of [K^+^] in DCT cells where [Cl^−^]_i_ is typically around 10–20 mM (Weinstein, 2005). Our model HEK cells behave like the DCT cells, with the downstream signaling targets of WNK4, the SPAK and NCC showing a corresponding decrease in their phosphorylation levels at [K^+^]_o_ of 5 mM. Although WNK1 has been reported to be the [K^+^] sensor in HEK cells (Terker et al., 2015) (due to its higher [Cl^−^]_i_ of >40 mM), the exogenously expressed WNK4 in our model cell probably functions as the primary sensor due to its much greater abundance over WNK1.

The abundance of both pSPAK and pNCC in our in vitro study showed an inverse relationship to the increase in [K]_o_, and interestingly, the pattern of pNCC reduction with [K]_o_ was comparable to the one observed in aldosterone untreated mice in in *vivo.* However, in the same study aldosterone‐treated mice showed a much steeper decline in pNCC abundance with an increase in plasma [K^+^] (Terker, Zhang, et al., 2016), suggesting a role for aldosterone in ameliorating the phosphorylation and thus activation of NCC at low plasma [K^+^].

Given NCC is the principal transporter for Na^+^ reabsorption in the DCT, it plays a crucial role in controlling blood pressure, and has been shown to be regulated by the hormone aldosterone indirectly via changes in plasma [K^+^] (van der Lubbe et al., 2012; Terker, Yarbrough, et al., 2016). Interestingly, a recent study revealed that aldosterone rapidly activates NCC, and that this effect is lost at high [K^+^] in ex vivo experiments. A membrane bound G protein‐coupled receptor, GPR30, mediated the effect of aldosterone on NCC in both the in vivo and ex vivo studies, and phosphoproteomics in combination with network analysis and further in vitro work showed the activation of the EGFR tyrosine kinase pathway by aldosterone through GPR30. Thus our understanding of the signaling pathways regulating NCC are increasingly detailed (Cheng et al., 2019). We have not investigated the aldosterone effects in our DCT‐like HEK cells, but this is an important avenue for further work. Our modified HEK cell system could function as a potential model to study other aspects of NCC regulation.

Considerable work has gone into the elucidation of NCC phosphorylation by the WNK/SPAK pathway whereas the molecular mechanisms controlling the dephosphorylation of NCC are still poorly understood. In one of our earlier in vitro studies in *Xenopus* oocytes, NCC activity was found to be regulated by Protein phosphatase 4 (PP4) through its interaction with a conserved N‐terminal amino acid threonine 58 of NCC, and this regulation was independent of WNK4 regulated NCC trafficking to the membrane (Glover, Mercier Zuber, Figg, & O'Shaughnessy, 2010). More recently, signaling pathways independent of the WNK4/SPAK cascade have been shown to regulate NCC phosphorylation (and therefore its activity) in the DCT (Penton et al., 2019). In vitro studies showed that protein phosphatase 1 (PP1) dephosphorylates NCC whereas the PP1 inhibitor calyculin A had the opposite effect, and protein kinase A (PKA)‐dependent phosphorylation of the PP1 inhibitor 1 abolished the PP1‐mediated dephosphorylation of NCC in ex vivo mouse kidney preparations (Penton et al., 2019). A comprehensive study of PP inhibitors in our cells has not been done to confirm if either PP1 or PP4 are important controllers of NCC dephosphorylation in our model HEK cells.

The calmodulin and calcineurin cascade has been shown to mediate NCC dephosphorylation after acute K^+^ loading, and this is independent of the anion (Shoda et al., 2017). It would be of interest to investigate the potential signaling pathways regulating NCC dephosphorylation which operate at physiologically relevant [K^+^], and we are using a proteomics approach in order to discover them.

In summary, we have shown using fluorescence‐based sensors that HEK cells expressing KCNJ10, Clc‐kb, and barttin are highly sensitive to changes in [K^+^]_o_ within the physiological range and also show the changes in intracellular [Cl^−^] that affect downstream WNK4/SPAK/NCC signaling. This confirms the hypothesized DCT model in which the transcellular flux of NaCl in the DCT is directly regulated by the membrane potential of the DCT cell and its direct coupling to the phosphorylation state of NCC through the Cl^−^ sensor WNK4.

## CONFLICT OF INTEREST

None declared.
